# A Pre-clinical Animal Model of *Trypanosoma brucei* Infection Demonstrating Cardiac Dysfunction

**DOI:** 10.1371/journal.pntd.0003811

**Published:** 2015-05-29

**Authors:** Charlotte S. McCarroll, Charlotte L. Rossor, Linda R. Morrison, Liam J. Morrison, Christopher M. Loughrey

**Affiliations:** 1 Institute of Cardiovascular and Medical Sciences, College of Medical, Veterinary and Life Sciences, University of Glasgow, Glasgow, United Kingdom; 2 Easter Bush Pathology, Royal (Dick) School of Veterinary Studies and The Roslin Institute, Easter Bush Campus, University of Edinburgh, Midlothian, United Kingdom; 3 Wellcome Trust Centre for Molecular Parasitology, Institute of Infection, Immunity and Inflammation, College of Medical, Veterinary and Life Sciences, University of Glasgow, Glasgow, United Kingdom; 4 Roslin Institute, Royal (Dick) School of Veterinary Studies, University of Edinburgh, Easter Bush, Midlothian, United Kingdom; New York University School of Medicine, UNITED STATES

## Abstract

African trypanosomiasis (AT), caused by *Trypanosoma brucei* species, results in both neurological and cardiac dysfunction and can be fatal if untreated. Research on the pathogenesis and treatment of the disease has centred to date on the characteristic neurological symptoms, whereas cardiac dysfunction (e.g. ventricular arrhythmias) in AT remains largely unstudied. Animal models of AT demonstrating cardiac dysfunction similar to that described in field cases of AT are critically required to transform our understanding of AT-induced cardiac pathophysiology and identify future treatment strategies. We have previously shown that *T*. *brucei* can interact with heart muscle cells (cardiomyocytes) to induce ventricular arrhythmias in *ex vivo* adult rat hearts. However, it is unknown whether the arrhythmias observed *ex vivo* are also present during *in vivo* infection in experimental animal models. Here we show for the first time the characterisation of ventricular arrhythmias *in vivo* in two animal models of AT infection using electrocardiographic (ECG) monitoring. The first model utilised a commonly used monomorphic laboratory strain, *Trypanosoma brucei brucei* Lister 427, whilst the second model used a pleomorphic laboratory strain, *T*. *b*. *brucei* TREU 927, which demonstrates a similar chronic infection profile to clinical cases. The frequency of ventricular arrhythmias and heart rate (HR) was significantly increased at the endpoint of infection in the TREU 927 infection model, but not in the Lister 427 infection model. At the end of infection, hearts from both models were isolated and Langendorff perfused *ex vivo* with increasing concentrations of the β-adrenergic agonist isoproterenol (ISO). Interestingly, the increased frequency of arrhythmias observed *in vivo* in the TREU 927 infection model was lost upon isolation of the heart *ex vivo*, but re-emerged with the addition of ISO. Our results demonstrate that TREU 927 infection modifies the substrate of the myocardium in such a way as to increase the propensity for ventricular arrhythmias in response to a circulating factor *in vivo* or β-adrenergic stimulation *ex vivo*. The TREU 927 infection model provides a new opportunity to accelerate our understanding of AT-related cardiac pathophysiology and importantly has the required sensitivity to monitor adverse cardiac-related electrical dysfunction when testing new therapeutic treatments for AT.

## Author Summary

African trypanosomiasis (AT) is a disease caused by the single-celled protozoan parasite *Trypanosoma brucei*. In humans, AT causes neurological problems including sleep disturbances, which give the disease its colloquial name of “sleeping sickness”. Much of the focus of AT research has been on the neurological deficits, but other major organs are also affected, including the heart. Previous studies in humans and animals with AT have identified heart abnormalities such as contractile dysfunction, ventricular arrhythmias and significant cardiac tissue inflammation post-mortem. The need for studies investigating AT-induced cardiac dysfunction is critically important. There is a growing cardiovascular disease (CVD) burden in the population of Sub-Saharan Africa and CVD is now considered the leading cause of death in those aged over 45. AT-induced cardiac dysfunction has the potential to compound poor outcome in patients with pre-existing CVD and those being treated with existing AT medications due to their inherent adverse cardiac effects. As a consequence, new treatments for AT require evaluation in appropriate pre-clinical animal models to ensure that they do not detrimentally alter cardiac function. This study established two rat models of AT infection in which an electrocardiogram (ECG) was used to study cardiac electrical function. The first model utilised a commonly used laboratory strain, *Trypanosoma brucei brucei* Lister 427, whilst the second model used a laboratory strain that demonstrates a similar chronic infection profile to clinical cases, *T*. *b*. *brucei* TREU 927. The TREU 927 infection model showed a significantly increased frequency of cardiac arrhythmias *in vivo*. The arrhythmias observed *in vivo* in the TREU 927 infection model were lost upon isolation of the heart *ex vivo*, but returned with the addition of the β-adrenergic agonist—isoproterenol (ISO). These data suggest that AT alters the myocardium in such a way as to increase the propensity for ventricular arrhythmias when in the presence of a circulating factor or β-adrenergic stimulation (stress). Importantly, this animal model has sufficient sensitivity to measure changes in the frequency of AT-induced ventricular arrhythmias. Therefore the model is of value for studies aiming to develop new treatment strategies to specifically minimise AT-related cardiac dysfunction or to assess the presence of adverse cardiac electrical abnormalities when testing new trypanocidal-based AT treatments.

## Introduction

Human African trypanosomiasis (HAT) is caused by the Trypanosoma brucei sub-species *T*. *b*. *gambiense* and *T*. *b*. *rhodesiense*, which are transmitted by the tsetse fly vector (*Glossina spp*.). Infection with these parasites leads to both neurological and cardiac dysfunction and can be fatal if untreated. The rate of disease progression and severity of clinical signs is dependent upon a number of factors including parasite species and strain. Whilst the neurological-related pathogenesis of African Trypanosomiasis (AT) infection has been an understandable research focus leading to its extensive characterisation [1–3], the pathogenesis associated with cardiac dysfunction is poorly understood. A better understanding of the cardiac-related pathogenesis of the disease is required because it is unknown: 1) how AT infection leads to cardiac pathology; 2) whether infection level or stage alters the severity and progression of cardiac dysfunction; 3) if the toxicity of drugs used to treat HAT could be exacerbated by AT-induced cardiac pathology; and 4) if direct treatment of the cardiac pathology can improve overall patient outcome.

HAT manifests clinically in two stages: Stage I, the haemolymphatic stage; and Stage II, the meningo-encephalitic stage where the parasites extravasate into organs including the brain [4–6] and heart [7;8]. Post-mortem studies clearly demonstrate that cardiac pathology occurs in 70–100% of HAT patients [7;9] and in experimental animal models of the disease [10–14]. The observed cardiac pathologies include myocarditis [9] with mononuclear inflammatory cell infiltration and fibrosis [7;8], both of which can lead to ventricular dysfunction and heart failure. In a study of sixteen HAT deaths from *T*. *b*. *gambiense*, Adams *et al*. (1986) found that two of the deaths were the result of pulmonary oedema from cardiac failure [7]. Furthermore, radiographs from two separate studies demonstrated an enlargement of the cardiac silhouette to >50% of the thoracic diameter (a potential indicator of cardiac failure) in 34% (of 118 [15]) and 44% (of 25 [16]) of patients with HAT (reviewed in Blum *et al.* 2008 [17]). Echocardiography of HAT patients is not commonly assessed, but Tsala *et al*. (1988) in a study of 25 HAT patients, identified potential indicators of heart failure including right-ventricular dilatation in 64% of HAT patients and pericardial effusion in 12% [16]. Fouchet *et al*. (1968) [18] and Bertrand *et al*. (1974) [15] have both reported cardiac conduction abnormalities, including type I Atrio-Ventricular (AV) block in 3.7% and 14% of patients examined and type II AV block in 1% and 2.5% of patients, respectively [15;18;19]. Conduction abnormalities can lead to disturbances in heart rate and rhythm, leading to impaired delivery of blood to the tissues. Palpitations resulting from ventricular arrhythmias called ventricular premature complexes (VPCs) are indicators of heart dysfunction. HAT patients have significantly more palpitations than controls [19]. A number of studies demonstrated a clear involvement of the cardiac pathology in death [7;15;20;21], but because of the logistical difficulties of performing significant field studies in the regions involved, understanding the true extent to which this occurs in HAT is currently limited. A more recent large scale study that collected significant quantities of cardiac data from *T*. *b*. *gambiense* HAT patients observed a high prevalence of cardiac electrical abnormalities in ~55% of 406 stage I HAT patients and ~71% of 60 stage II HAT patients [19;22]. This study also demonstrated a significant proportion of patients with: (i) prolonged QT interval which can lead to fatal arrhythmias; and (ii) increased levels of the peptide NT- proBNP, an indicator of left ventricular dysfunction [19]. The extent of electrical abnormalities reported in patients with HAT in the study by Blum *et el* (71%) [19] is in striking agreement with the proportion of patients with cardiac pathology observed at post-mortem [7;9]. In addition to *T*. *b*. *gambiense* HAT patients, ECG abnormalities have also been identified in 55% (22 of 40) of patients with East African trypanosomiasis caused by *T*. *b*. *rhodesiense* (unclassified for stage I or II disease) [23].

Besides the inevitable inflammatory response to trypanosomes that occurs in heart tissues, we have previously demonstrated that *T*. *brucei* directly interact with heart muscle cells (cardiomyocytes) to induce ventricular arrhythmias (VPCs) in *ex vivo* hearts independent of a systemic immune/inflammatory response [24]. However, it is unknown whether the arrhythmias observed in rat hearts *ex vivo* are also present during infection *in vivo*.

The main aim of the current study was to develop an animal model of AT infection to assess whether ventricular arrhythmias—in particular VPCs—could be identified *in vivo* through electrocardiographic (ECG) assessment. The data suggest for the first time that AT infections create an arrhythmogenic substrate in the heart, which when triggered by circulating factor/s associated with β-adrenergic stimulation, increase the propensity for ventricular arrhythmias.

## Methods

### Preparation of Trypanosomes

*T*. *b*. *brucei* Lister 427 and *T*. *b*. *brucei* TREU 927 were grown separately for one passage in ICR mice for 2–3 days to adapt them to *in vivo* conditions, from being culture adapted in the case of *T*. *b*. *brucei* Lister 427 [25] and from cryopreservation for *T*. *b*. *brucei* TREU 927. When parasitaemia reached 1 x 10^8^ parasites.mL^-1^ [26] the mice were euthanased and blood collected in Carter’s balanced salt solution (CBSS; mM; 25 HEPES, 120 NaCl, 5.4 KCl, 0.55 CaCl_2_, 0.4 MgSO_4_, 5.6 Na_2_PO_4_, 11.1 glucose, pH 7.4) containing 100 U/ml heparin. The trypanosomes were diluted to 1 x 10^5^ parasites in a 200 μL volume of CBSS (approx. 1:200 dilution from 1 x10^8^ parasites.mL^-1^) under sterile conditions. The 200 μL parasite suspension was prepared in a 1 mL syringe for injection. Matching volumes of CBSS were prepared as control injections. All mice and rat animal procedures were approved by the University of Glasgow Ethical Review Panel and licensed by the Home Office, UK (Project Licence Number 600/4503).

### Animals

The care and use of animals was in accordance with the UK government Animals (Scientific procedures) Act 1986 (ASPA). Animals used were adult male Wistar rats (250–300g) with a 7 day acclimatisation period upon delivery with a 12 hr light/dark cycle. Animals were kept at the Cardiovascular Research Unit, University of Glasgow in a dedicated room licensed under the Specified Animal Pathogens (Scotland) Order, 2009 (SAPO).

### ECG Acquisition for the Lister 427 Infection Model

Anaesthesia was induced by placing the animal in a pre-filled induction chamber with 5% isoflurane (Isoflo, Abbot Laboratories, USA) in 100% O_2_ at 1 L.min^-1^ until loss of righting and flexor withdrawal reflexes. The rats were then maintained on isoflurane delivered *via* facemask at 1–1.5% isoflurane in 1 L.min^-1^ O_2_. The lead II ECG was recorded *via* the placement of sterile intradermal electrodes. The placement sites on the rat were caudal aspects of the left and right carpi and the medial aspects of left and right crura. To ensure reproducibility for the same rats and between rats, all animals were positioned identically. The ECG was recorded for 15 min with an IWX228 bioamplifier and LabScribe 2 software (iWorx) at a sampling rate of 2.0 KHz. Rats were then injected *via* the intraperitoneal route with *T*. *b*. *brucei* Lister 427 or vehicle and then recovered.

### Data Acquisition and Analysis for the Lister 427 Infection Model

The ECG data from the last 1 min of the 15 min period collected on both day 0 and day 4 were averaged using the advanced ECG analysis module (iWorx) and exported to Origin6.1 (OriginLab) for RR interval, heart rate, PR interval and QT interval measurement, with correction for heart rate using the Framingham method [27]. The whole trace was manually assessed for ventricular premature complexes (VPCs) as defined by the Lambeth Conventions [28]; specifically complexes that were premature, without a well-defined P wave and had a wide and bizarre shape.

### Parasitaemia Measurements

Parasitaemia levels were measured daily by microscopy of blood from superficial venepuncture of the lateral tail vein, as previously described [26]. A parasitaemia level exceeding 5.0 x 10^8^ parasites.mL^-1^ for more than two consecutive days was set as a cut-off point for the welfare of the animals. No animal exceeded this level during the study. Day 4 of the Lister 427 model was selected as the end point based on prior experience with this trypanosome strain in the rat infection model in terms of the level of parasitaemia and its impact on the health of the animals and in order to avoid sudden deaths.

### Telemetry Probe Implantation for the TREU 927 Infection Model

For the longer TREU 927 infection model, CA-F40 biopotential recording devices (Data Sciences International) were implanted into rats to measure the ECG in conscious animals. This allowed the recording and analysis of ECG data without the potential cardiovascular depressive effects of isoflurane [29;30]. Briefly, adult male Wistar rats (250–300 g) were anaesthetised with isoflurane delivered in 100% O_2_. Animals were positioned in ventral recumbency on a heated pad. Peri-operative analgesia of 5.0 mg.kg^-1^ carprofen (Rimadyl, Pfizer Animal Health) was administered in 5.0 mL of 0.9% sterile saline subcutaneously. The telemetry device was implanted subcutaneously in the dorsal thoracic region. Tracts were tunnelled under the skin from the implant site to the right pectoral and xyphoid regions ventrally. The insulation was removed from the distal 5.0 mm of each ECG lead, which were then affixed to the layer of muscle at each point under the skin with 1.5 metric nylon suture (Johnson & Johnson). Exposed ends of the leads were re-covered with the removed insulating material. The surgical incisions were closed with 1.5 metric polyglactan 910 (Vicryl, Johnson & Johnson).

### Trypanosome Infections for TREU 927 Infection Model

The animals were allowed to recover from the surgical procedure for 1 week before they were infected with 1.0 x 10^5^ *T*. *b*. *brucei* TREU 927 in 200 μL CBSS *via* intraperitoneal injection. Control rats were injected with the same volume of CBSS. Parasitaemia levels by superficial tail venepuncture were assessed as previously described.

### Data Acquisition and Analysis for TREU 927 Infection Model

The implanted probes were activated magnetically and data was collected from receiver pads underneath the animals’ usual cages. Telemetry signals were relayed *via* a data exchange matrix to a computer loaded with the acquisition software Dataquest™ OpenART v4.2 (Data Sciences International). Raw ECG data was collected at a 2.0 kHz sampling frequency. Files were exported to Ponemah v4.8 (Data Sciences International) for analysis. ECG sections of 30 min were averaged and assessed for RR interval, heart rate, PR intervals, and QT intervals corrected for heart rate using the Framingham method (QTc = QT + 0.154 x (1-RR)). A one hour section of trace from day 3, 6, 9 and the end of the final day of the protocol—to enable analysis of time points corresponding with peak and trough parasitaemias—was assessed for arrhythmias using the Lambeth Conventions as described above and compared to a one hour section of trace after recovery but prior to infection (day 0).

### Organ Harvest

Both control and infected animals for each model were sacrificed by cervical dislocation while under a brief exposure of low dose 1% isofluorane. Heart, liver and spleen were removed, organ mass recorded and compared to the tibial length defined as the length from lateral femoral epicondyle proximally to the lateral malleolus distally to control for individual size and mass.

### Langendorff Perfusion of *Ex Vivo* Hearts

The hearts removed from both groups (Lister 427 infection model and TREU 927 infection model, plus controls) were immersed in ice-cold Tyrodes solution (mM); 116 NaCl, 20 NaHCO_3_, 0.4 Na_2_HPO_4_, 1.0 MgSO_4_-7H_2_O, 4.0 KCl and 11.0 D-glucose. The solution was bubbled with 95% O_2_ / 5% CO_2_ for 15–20 min to oxygenate and buffer before CaCl_2_ was added to a concentration of 1.8 mM. Extraneous tissue was carefully dissected away to reveal the aorta. The hearts were blotted dry and quickly weighed before cannulation to a Langendorff perfusion apparatus. The hearts were perfused with the above Tyrodes solution at 10 mL.min^-1^ and immersed in a water-jacketed chamber filled with Tyrodes solution at 37°C. Electrodes were placed in the chamber in close approximation with the right atrium for the negative electrode and the apex of the left ventricle for the positive electrode and the pseudo-ECG recorded. Hearts were perfused for a period of 15 min (steady state) followed by 15 min periods each with the β-adrenergic agonist isoproterenol at concentrations of 100 nM, 1 μM, 10 μM and 100 μM.

### Langendorff Data Analysis

ECG data were collected using the ETH-256 bioamplifier (iWorx) and LabChart 7 (ADInstruments) software at a sampling rate of 2.0 kHz. The ECG from the last 5 min of each 15 min period was averaged using the advanced ECG analysis module of the programme and the RR interval, heart rate, PR interval and the QT interval with correction for heart rate using the Framingham method [27] were measured. Traces were exported to Origin6.1 (OriginLab). The entirety of the traces was manually assessed for arrhythmic events according to the Lambeth Conventions [28] and recorded as the frequency.min^-1^.

### Histology of Hearts

After Langendorff perfusion, whole hearts were fixed in 10% neutral buffered formalin and sectioned to include a transverse section of the apex and one longitudinal section of the base (to include the atria) and the apex (3 sections from each heart). These were then routinely processed to paraffin wax and serial sections (5 μm) were stained with haematoxylin and eosin (H&E) for histopathological evaluation of inflammation and Picrosirius red for fibrosis. Selected sections were stained with Giemsa to highlight parasitic organisms. The sections were then semi-quantitatively scored and assigned a score of 0–3 for degree of inflammation and fibrosis. The scores of 0–3 were based on the findings of no pathology, mild, moderate and severe changes respectively. In addition, the presence (1) or absence (0) of parasites and their location (e.g. interstitial) was noted.

### Statistics

For the *in vivo* Lister 427 infection model, the mean ± SEM data from the last 1 min of ECG trace for the same animal from day 0 and day 4 were compared with a paired Student’s T-test. Comparisons of *in vivo* ECGs between control and infected animals were conducted with a two-sample Student’s T-test. For the *in vivo* TREU 927 infection model, mean ± SEM ECG telemetry data were taken from the last 30 min of ECG trace from day 0 and day 11 and compared within each animal by paired Student’s T-test. Comparisons between control and infected animals were performed by two-sample Student’s T-test. Arrhythmia data for the TREU 927 infection model were taken from 1 hr long traces at days 0, 3, 6, 9 and the end and compared to day 0 by ANOVA. Mean ± SEM ECG data was acquired from the average of the last 5 min of each 15 min section of trace for each concentration of ISO for the *ex vivo* Langendorff perfused hearts of both the Lister 427 and TREU 927 infection models and analysed using ANOVA. *P*<0.05 was taken to be statistically significant.

## Results

### *In Vivo* ECG Parameters for *T*. *b*. *brucei* Lister 427 Infections

**Fig 1:**
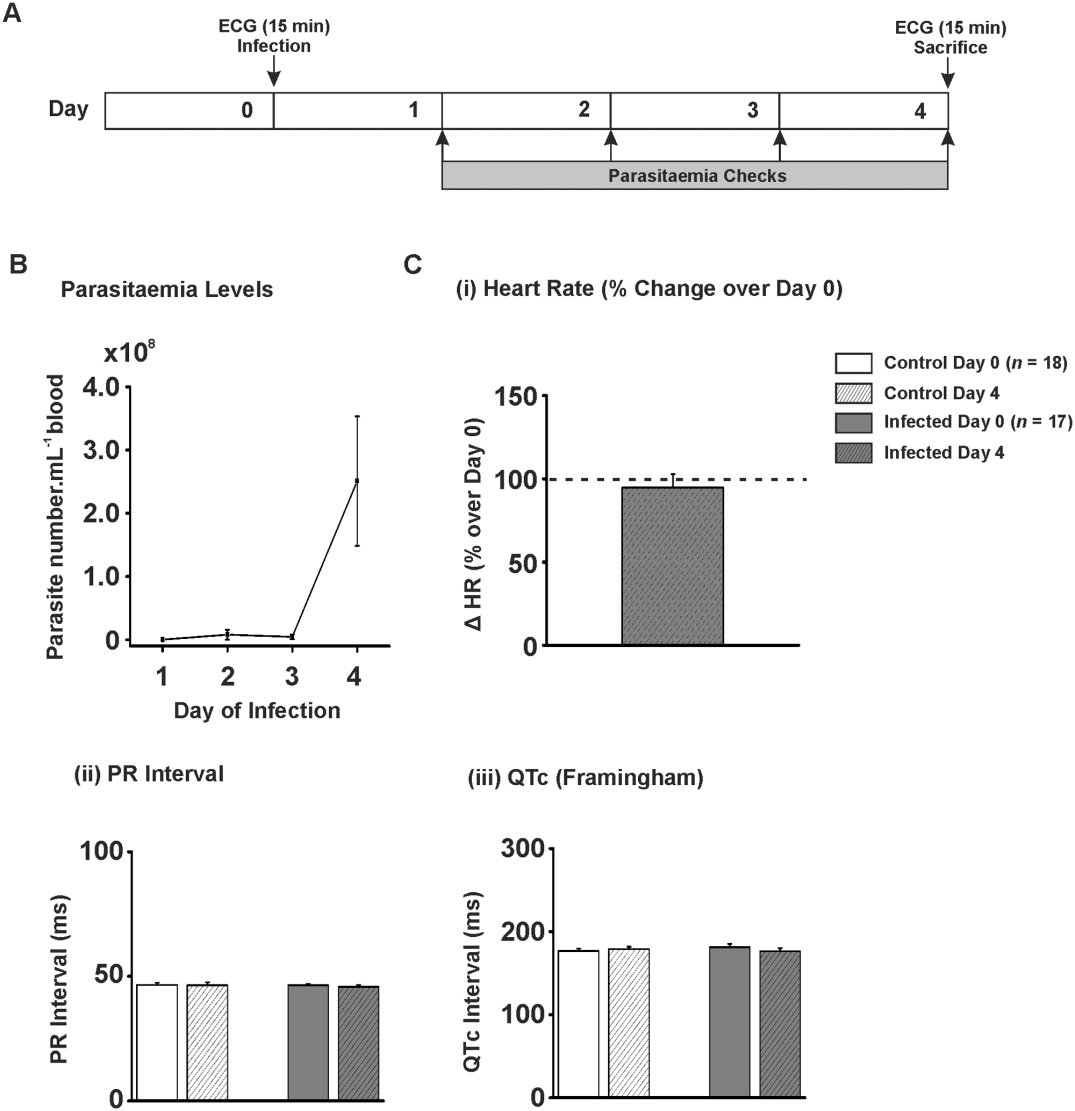
*In vivo* ECG parameters for T. b. brucei Lister 427 infection. A) Protocol used for *in vivo T*. *b*. *brucei* Lister 427 infection model. ECGs were recorded prior to injection with trypanosomes or control vehicle (CBSS). ECGs were recorded again on day 4 prior to sacrifice. B) Parasitaemia levels for *T*. *b*. *brucei* Lister 427 infection model. Numbers expressed as parasites.mL^-1^ of blood x 10^8^. C) (i) Mean percentage change in heart rate on day 4 over heart rate on day 0. C) (ii-iii) Mean data for PR interval and QT interval corrected for heart *via* Framingham method.

The average ECG traces from 15 min periods from days 0 and 4 from anaesthetised rats in both infection and control groups were analysed for heart rate (HR), PR interval and QT interval (Fig 1A). The Lister 427 infection model was of short duration—animals had to be euthanased on welfare grounds at 4 days post-inoculation. The relatively brief infection period was due to parasitaemia of the infected rats demonstrating exponential growth (rising to 2.51 x 10^8^ ± 1.02 x 10^8^ parasites.mL^-1^ on day 4; Fig 1B), as was anticipated with this monomorphic trypanosome strain. There was no significant difference in the HR in the control animals between days 0 and 4 (99.8 ± 8.6% of day 0; 249 ± 21 *vs.* 248 ± 21 bpm; Day 0 *vs*. Day 4; *P*>0.05), nor was there a significant difference in the infected animals (94.8 ± 7.9% of day 0; 238 ± 19 *vs.* 226 ± 19; Day 0 *vs*. Day 4; *P*>0.05; Fig 1C(i)). There was no significant difference in PR interval for the control rats (46.6 ± 0.8 *vs.* 46.4 ± 1.2 ms; Day 0 *vs*. Day 4; *P*>0.05; Fig 1C(ii)), nor for the infected rats (46.4 ± 0.6 *vs.* 45.8 ± 0.8 ms; Day 0 *vs*. Day 4; *P*>0.05; Fig 1C(ii)). There was also no significant difference in QTc for control (176.6 ± 3.0 *vs.* 179.1 ± 2.8 ms; Day 0 *vs*. Day 4; *P*>0.05; Fig 1C(iii)) or infected animals (181.3 ± 3.9 *vs.* 176.2 ± 3.9 ms; Day 0 *vs*. Day 4; *P*>0.05; Fig 1C(iii)). During the 15 min periods of ECG for both the start and end of the study protocol no ventricular arrhythmic events were observed.

### *Ex Vivo* Langendorff Perfused Heart Pseudo-ECG Parameters for *T*. *b*. *brucei* Lister 427 Infections

**Fig 2:**
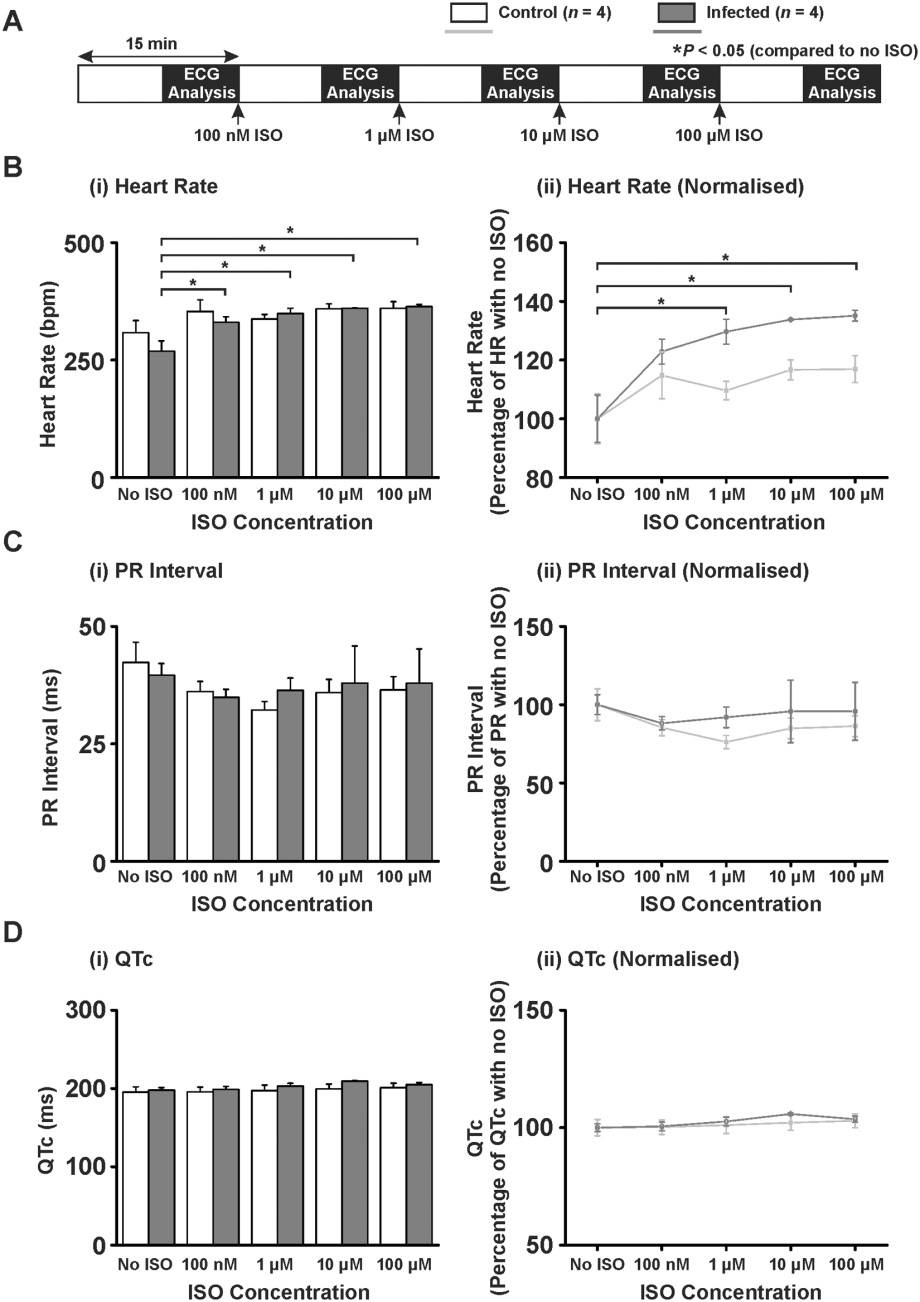
*Ex vivo* Langendorff ECG parameters for T. b. brucei Lister 427 infection. A) Protocol used in Langendorff perfusion experiments. Increasing concentrations of isoproterenol were added every 15 min and the ECG recorded throughout. B) (i) Mean ± SEM heart rate data (values also in Table 1) and (ii) heart rate normalised to no ISO. C) (i) Mean ± SEM PR interval data (values also in Table 1) and (ii) PR interval normalised to no ISO. D) (i) Mean ± SEM QT interval corrected for heart rate (QTc) (values also in Table 1) and (ii) QT interval corrected for heart rate normalised to no ISO. Statistics performed on raw data by paired Student’s T-test to no ISO, *P*<0.05 taken as significant denoted by *.

An ECG was performed on perfused hearts isolated from infected animals to determine: (1) whether cardiac electrical activity was altered in the absence of a systemic autonomic nervous response or circulating factors and (2) the effect of β-adrenergic stimulation on ECG parameters using isoproterenol (ISO) at increasing doses (100 nM, 1 μM, 10 μM and 100 μM) every 15 min to simulate a stress response (Table 1 and Fig 2A).

**Table 1:** *T*. *b*. *brucei* Lister 427 *ex vivo* Langendorff parameters.

ISO (μM)	Heart Rate (bpm)*			PR Interval (ms)*			QTc (Framingham (ms))		
	Control (*n* = 4)	Infected(*n* = 4)	*P* value	Control (*n* = 4)	Infected(*n* = 4)	*P* value	Control (*n* = 4)	Infected (*n* = 4)	*P* value
0	308 ± 26	269 ± 22	0.295	42.3±4.3	39.6±2.5	0.605	195.3±6.8	197.8±3.3	0.750
0.1	353 ± 25	331 ± 11	0.431	36.1±2.2	34.9±1.7	0.673	195.7±6.0	198.9±3.7	0.665
1.0	337 ± 10	349 ± 11	0.472	32.2±1.8	36.4±2.6	0.239	197.4±7.0	203.0±3.6	0.502
10.0	359 ± 10	360 ± 1	0.963	35.9±2.8	37.9±7.9	0.767	199.4±6.2	209.3±0.8	0.343
100.0	360 ± 14	363 ± 4	0.880	36.5±2.8	37.9±7.3	0.826	201.0±5.8	204.9±2.6	0.685

*bpm; beats per minute, ms; milliseconds

When hearts were isolated and perfused *ex vivo*, no ECG parameters (HR, PR, QTc intervals) were significantly different between control and infected animals (Timepoint 0; Table 1 and Fig 2). HR of control animal hearts (normalised to no ISO) demonstrated a non-significant increase to 117% of no ISO level (100 ± 8.0 *vs.* 117 ± 5.0%; no ISO *vs.* 100 μM ISO; *P*>0.05; Fig 2B(i and ii) and Table 1). However, hearts from infected animals demonstrated a significant increase in HR to 135% of HR with no ISO (100 ± 8.0 *vs.* 135 ± 2.0%; no ISO *vs.* 100 μM ISO; *P*<0.05; Fig 2B(i and ii) and Table 1). PR or QTc interval (raw data and data normalised to no ISO) showed no significant difference between control or infected animals (Table 1 and Fig 2C and 2D respectively).

### Parasitaemia Levels in *T*. *b*. *brucei* TREU 927 Infections

**Fig 3:**
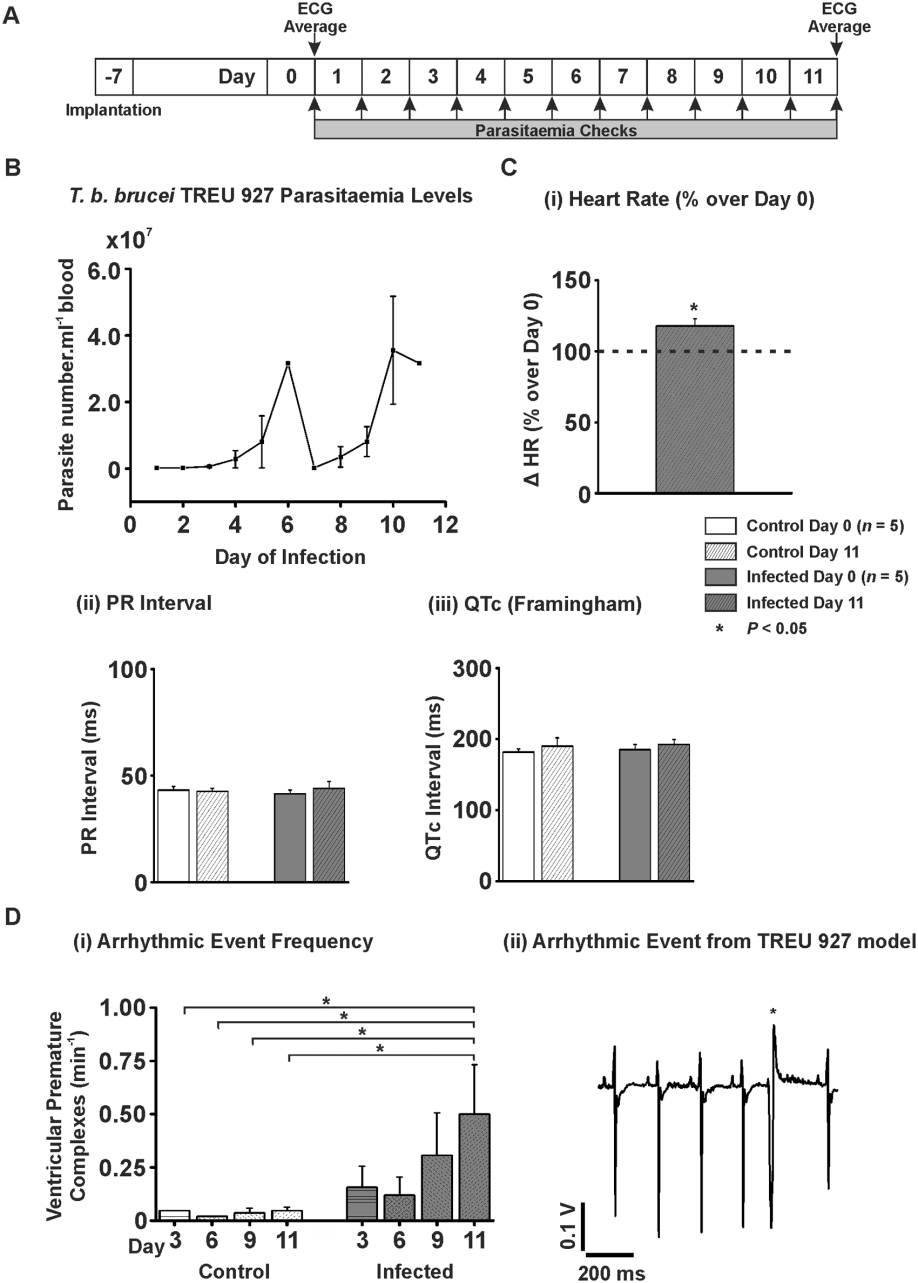
*In vivo* ECG parameters for T. b. brucei TREU 927 infection. A) Protocol used for *in vivo T*. *b*. *brucei* TREU 927 infection model. B) Parasitaemia levels for *T*. *b*. *brucei* TREU 927 infection model. Numbers expressed as parasites.ml^-1^ of blood x 10^7^ (*n* = 5). C) (i) Mean percentage change in heart rate on day 11 over heart rate on day 0. C) (ii-iii) Mean data for PR interval and QT interval corrected for heart *via* Framingham method from average traces taken prior to injection and end of the protocol. D) (i) Mean data for frequency of VPCs per min at days 3, 6, 9 and at the end of the model. D) (ii) Example trace from a TREU 927 infected animal.

A limitation of the use of *T*. *b*. *brucei* Lister 427 *in vivo* is that the parasite continues to divide exponentially within the host’s bloodstream until the death of the host, typically after approximately 4 days in the rat model. Lister 427 is a monomorphic strain of *T*. *b*. *brucei*, meaning it remains as the mitotically dividing long slender form [31]. While this continual growth makes Lister 427 a useful *in vitro* model organism, it does not mimic the fluctuating parasitaemia and more chronic infections [32] observed in human patients and animals infected with trypanosomes, which is due to the pleomorphism of non-laboratory adapted trypanosomes. Wild-type pleomorphic trypanosomes such as TREU 927 terminally differentiate to short stumpy forms under a density-dependent trigger [33] in preparation for transmission to the insect vector [34;35]. The animals were monitored for health and their parasitaemia was measured daily for the duration of the study (Fig 3A). The parasitaemia in the *T*. *b*. *brucei* TREU 927 infected animals followed the classical fluctuating pattern with a first peak parasitaemia of 3.16 x 10^7^ ± 2.51 x 10^5^ parasites.mL^-1^ of blood on day 6, before becoming undetectable by microscopy, followed by a second peak of 3.56 x 10^7^ ± 1.62 x 10^7^ parasites.mL^-1^ blood on day 10 (Fig 3B).

### *In Vivo* ECG Parameters for *T*. *b*. *brucei* TREU 927 Infections

The ECG was recorded in both control rats and rats infected with *T*. *b*. *brucei* TREU 927 *via* biopotential recording telemeters. Average ECG traces were analysed from day 0 and day 11 (Fig 3A). HR tended to decrease, but not significantly, in the control animals (404 ± 18 *vs.* 348 ± 12 bpm; Day 0 *vs*. Day 11; *P*>0.05). However, there was a significant increase in heart rate for TREU 927 infected animals of 117.8 ± 5.0% of day 0 (351 ± 22 *vs.* 413 ± 18 bpm; Day 0 *vs*. Day 11; *P*<0.05; Fig 3C(i)). There was no significant difference in PR interval for control (43.2 ± 1.7 *vs.* 42.6 ± 1.5 ms; Day 0 *vs*. Day 11; *P*>0.05; Fig 3C(ii)) or infected animals (41.5 ± 1.7 *vs.* 44.1 ± 3.2 ms; Day 0 *vs*. Day 11; *P*>0.05; Fig 3C(ii)). When the QT interval was corrected for HR using the Framingham method there was no significant difference for control animals (181.9 ± 4.4 *vs.* 190.1 ± 11.8 ms; Day 0 *vs*. Day 14; *P*>0.05; Fig 3C(iii)) or infected animals (185.2 ± 7.2 *vs.* 192.6 ± 6.9 bpm; Day 0 *vs*. Day 14; *P*>0.05; Fig 3C(iii)). The frequency of arrhythmic events including VPCs, did not significantly change in control animals (0.027 ± 0.019 *vs.* 0.047 ± 0.017 VPC.min^-1^; Day 0 *vs*. Day 11; *P*>0.05; Fig 3D(i)). However, during progression of infection with *T*. *b*. *brucei* TREU 927, when the arrhythmia frequency was examined at 3 day intervals to take into account both peaks and troughs of parasitaemia, the frequency increased over the course of the model to a final significant increase of 442% of Day 0 levels at Day 11 (0.113 ± 0.039 *vs.* 0.500 ± 0.234 VPC.min^-1^; Day 0 *vs*. Day 11; *P*<0.05; Fig 3D(i and ii)). Arrhythmia frequency did not parallel parasitaemia level since the arrhythmia frequency did not concomitantly reduce with the parasitaemia when examined at Day 9 (0.307 ± 0.199 VPC.min^-1^; Day 9; Fig 3D(i)).

### *Ex Vivo* Langendorff Perfused Heart Pseudo-ECG Parameters for *T*. *b*. *brucei* TREU 927 Infections

**Fig 4:**
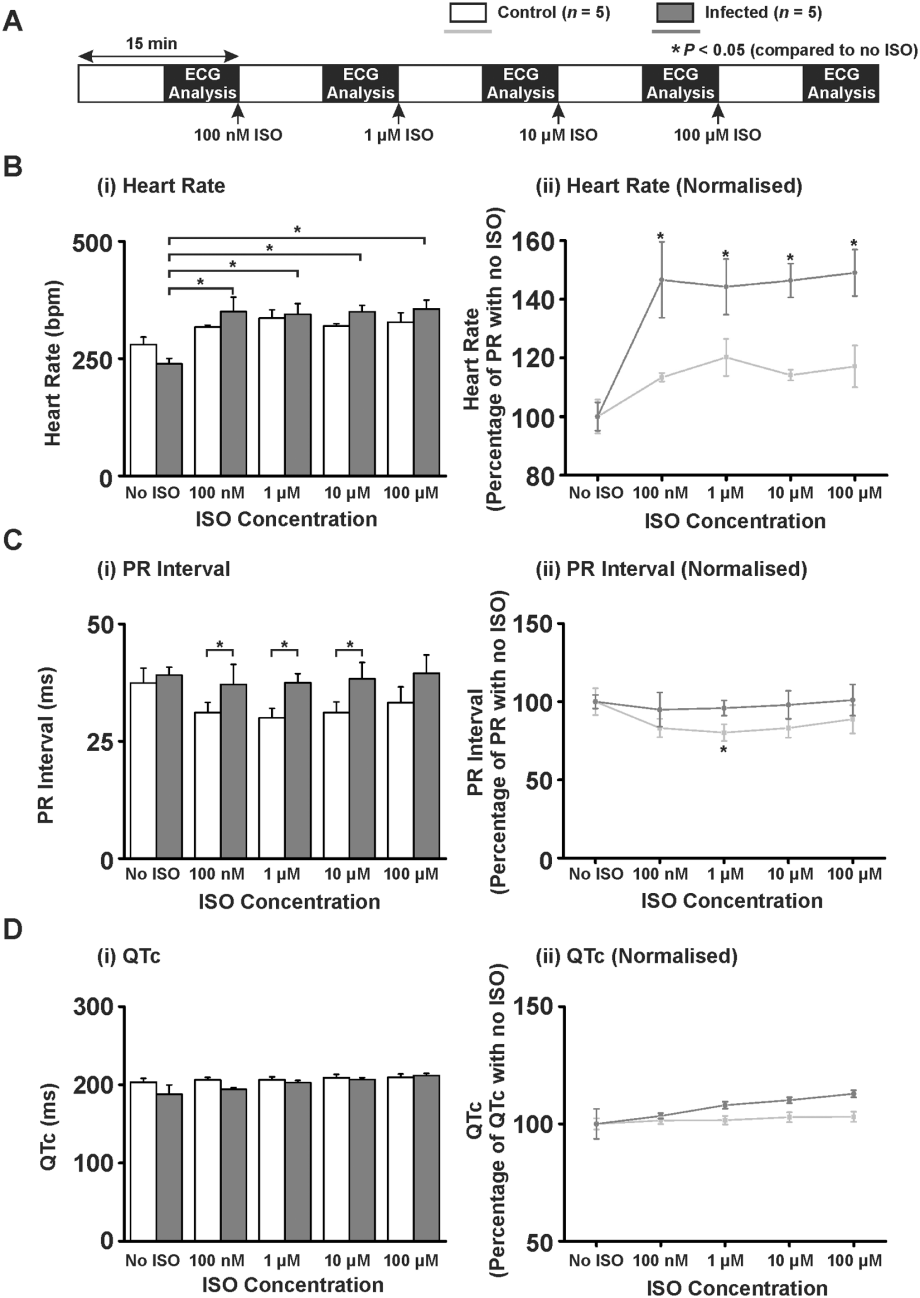
*Ex vivo* Langendorff ECG parameters for T. b. brucei TREU 927 infection. A) Protocol used in Langendorff perfusion experiments. Increasing concentrations of isoproterenol were added every 15 min and the ECG recorded throughout. B) (i) Mean ± SEM heart rate data (values also in Table 2) and (ii) Heart rate normalised to no ISO. C) (i) Mean ± SEM PR interval data (values also in Table 2) and (ii) PR interval normalised to no ISO. D) (i) Mean ± SEM QT interval corrected for heart rate (QTc) (values in also Table 2) and (ii) QT interval corrected for heart rate normalised to no ISO. Statistics performed on raw data by paired Student’s T-test to no ISO, *P*<0.05 taken as significant denoted by *.

As with the Lister 427 infection model, hearts were isolated from the TREU 927 infection model animals and an ECG performed in the presence and absence of ISO (Table 2 and Fig 4A). When hearts were isolated and perfused *ex vivo*, no ECG parameters (HR, PR, QTc intervals) were significantly different between control and infected animals (Timepoint 0; Table 2). The control animal HR (normalised to no ISO), demonstrated a significant increase to 117% of no ISO level (100 ± 6.0 *vs.* 117 ± 7.0%; no ISO *vs.* 100 μM ISO; *P*>0.05; Fig 4B(i and ii) and Table 2) compared to an increase to 149% of no ISO level for infected hearts (100 ± 5.0 *vs.* 149 ± 8.0; no ISO *vs.* 100 μM ISO; *P*<0.05; Fig 4B(i and ii) and Table 2). The PR interval (normalised to no ISO), demonstrated a significant difference between control and infected hearts but only at 1 μM ISO (100 ± 9.0 *vs.* 80.2 ± 4.9%; no ISO *vs.* 1μM ISO; *P*<0.05; Fig 4C(i & ii) and Table 2). No significant changes in the QTc (when normalised to no ISO) were observed (Fig 4D(i & ii) and Table 2).

**Table 2:** *T*. *b*. *brucei* TREU 927 *ex vivo* Langendorff parameters.

ISO (μM)	Heart Rate (bpm)*			PR Interval (ms)*			QTc (Framingham (ms))		
	Control (*n* = 5)	Infected(*n* = 5)	*P* value	Control (*n* = 5)	Infected(*n* = 5)	*P* value	Control (*n* = 5)	Infected (*n* = 5)	*P* value
0	280 ± 16	239 ± 11	0.073	37.4±3.2	39.1±1.7	0.665	203.3±4.9	187.8±12.0	0.265
0.1	317 ± 4	350 ± 31	0.321	31.1±2.2	37.1±4.3	0.245	206.3±3.2	194.2±2.2	0.015
1.0	336 ± 18	345 ± 23	0.780	30.0±2.0	37.5±1.9	0.028	206.5±2.8	202.8±2.8	0.451
10.0	319 ± 5	350 ± 14	0.073	31.1±2.3	38.3±3.5	0.118	209.1±4.3	206.8±2.4	0.655
100.0	328 ± 20	356 ± 19	0.334	33.2±3.4	39.5±3.9	0.252	209.5±4.4	211.9±2.8	0.657

*bpm; beats per minute, ms; milliseconds

### *T*. *b*. *brucei* TREU 927 Infected *Ex Vivo* Hearts Show Increased Frequency of Arrhythmias in the Presence of Isoproterenol

**Fig 5:**
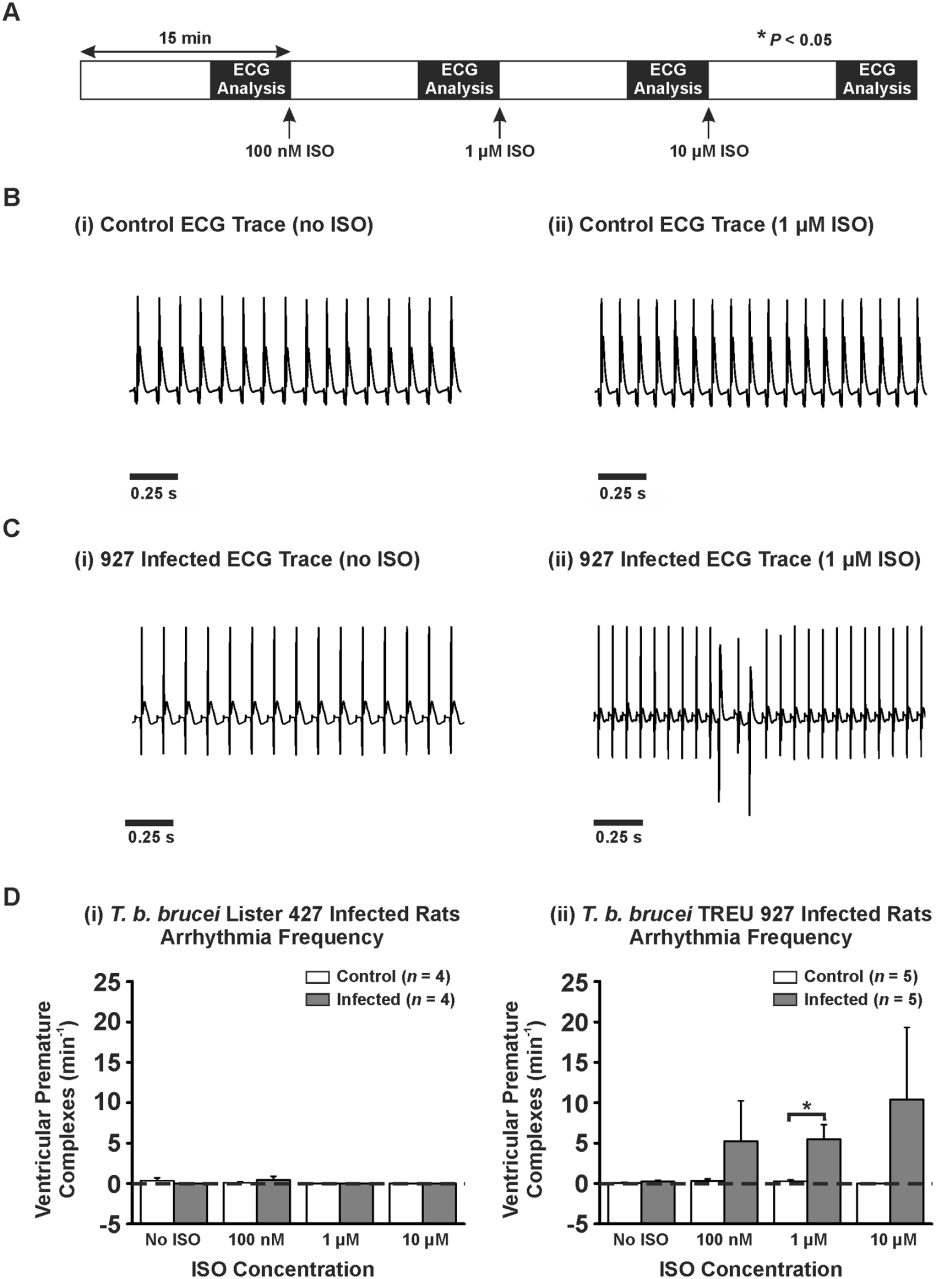
Arrhythmic events in Langendorff perfused infected and control hearts. A) Protocol used for Langendorff perfusion experiments. Hearts were perfused at a rate of 10 mL.min^-1^ for 15 min steady state followed by 15 min with increasing concentrations of isoproterenol (100 nM, 1 μM, and 10 μM). B-C) (i-ii) Example pseudo-ECGs from Langendorff perfused heart from TREU 927 infected animal and corresponding control. D) Mean ± SEM for frequency of ventricular premature complexes (VPCs) for (i) *T*. *b*. *brucei* Lister 427 and (ii) *T*. *b*. *brucei* TREU 927.

The pseudo-ECGs from the Langendorff perfused hearts from both models were also assessed for the frequency of VPCs with and without the presence of ISO (Fig 5A). There was no significant increase in VPC frequency in control hearts or the Lister 427 infection model hearts (Fig 5B and 5D(i)) both in the absence and presence of ISO. However, the TREU 927 infection model hearts demonstrated an increase in VPC frequency in the presence of ISO compared with control hearts (significant at 1 μM) (0.24 ± 0.16 *vs.* 5.24 ± 4.99 *vs.* 5.48 ± 1.83 (*P*<0.05) *vs.* 10.4 ± 8.9 VPC.min^-1^; no ISO *vs.* 100 nM *vs.* 1 μM *vs.* 10 μM ISO; Fig 5C and 5D(ii)) but not in the absence of ISO.

### Histology of *T*. *b*. *brucei* Infected Hearts

**Fig 6:**
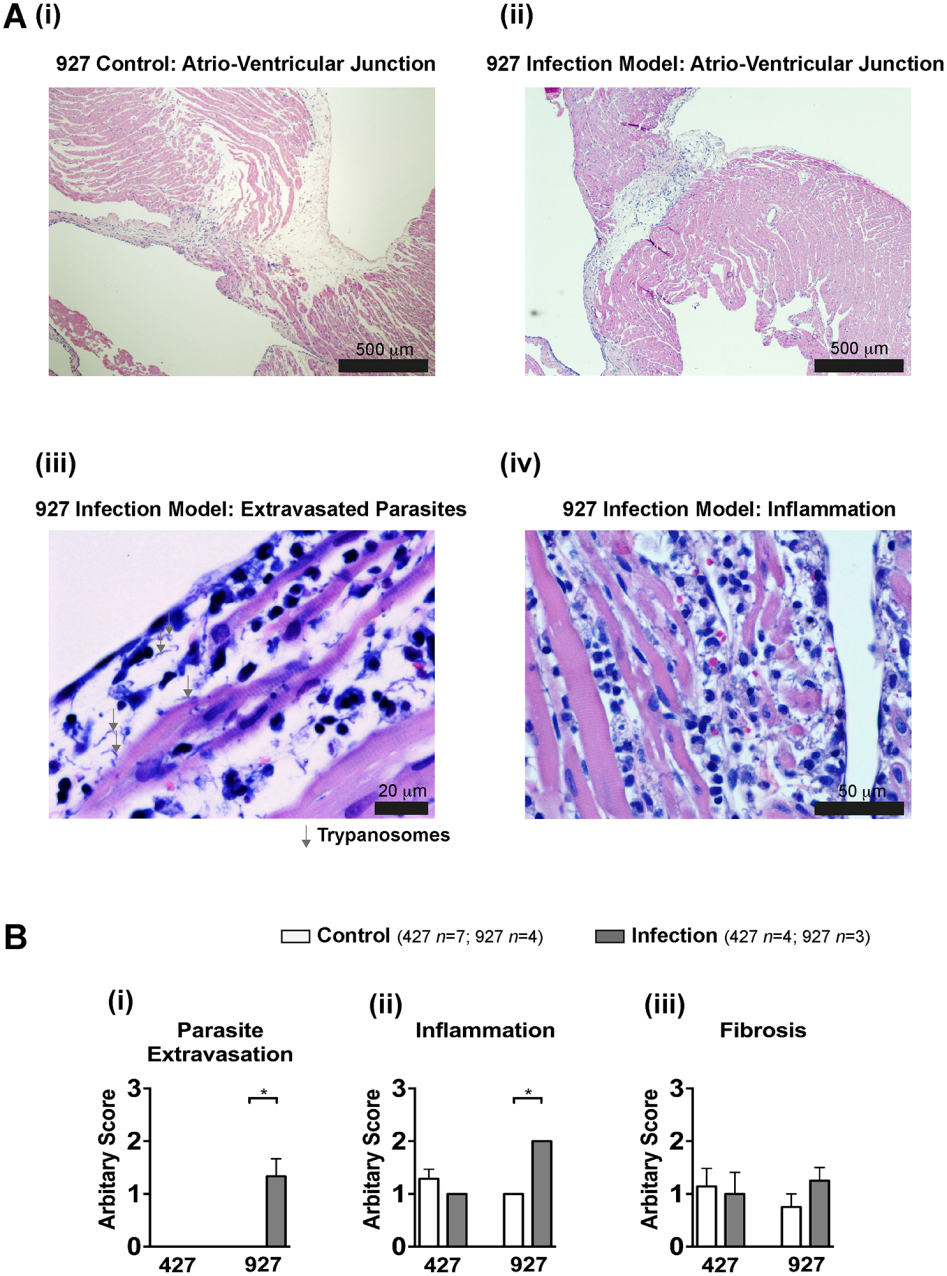
Histology of infected hearts. A) Atrio-ventricular region from (i) control and (ii) TREU 927 infected hearts (H&E). Higher magnification demonstrating (iii) parasite extravasation (Giemsa) and (iv) inflammation (H&E). B) Mean ± SEM of semi-quantitative pathological scoring (0–3; see methods) for: (i) parasite extravasation, (ii) inflammation and (iii) fibrosis.

Histopathological evaluation of the hearts from both control (Fig 6A(i)) and infection models (Fig 6A(ii)) subsequent to Langendorff perfusion revealed the presence of parasites within the interstitium (parasite extravasation) in close proximity to cardiomyocytes in 3 out of 3 (100%) of the TREU 927 infection model hearts but in 0 out of 4 hearts (0%) from the Lister 427 infection model and 11 control hearts (Fig 6A(iii) and 6B(i)). The level of inflammation was higher in hearts from the TREU 927 infection model compared to control and Lister 427 infected hearts (Fig 6A(iv) and 6B(ii)). There was no significant change in the degree of fibrosis between any group of hearts (Fig 6(iii)).

### Organ Weights in the Lister 427 and TREU 927 Infection Models

When animals were sacrificed, the mass/tibial length ratio of the heart, liver and spleen were recorded. Heart and liver mass/tibial length ratios were not significantly different between infected animals and controls for either the Lister 427 infection model or the TREU 927 infection model (see S1A(i–v) Fig and S2(i–iii) Fig However, the spleen mass/tibial length ratios were significantly increased in both the Lister 427 infection model by 199% (13.3 ± 0.4 *vs.* 26.5 ± 3.4 mg.mm^-1^; control *vs*. infected; *P*<0.05; S1(v) and S1B Fig) and the TREU 927 infection model by 292% (17.2 ± 1.6 *vs.* 50.2 ± 7.5 mg.mm^-1^; *P*<0.05; S2A(iv) Fig).

## Discussion

### *In Vivo* ECG Parameters for *T*. *b*. *brucei* Lister 427 Infections

We have previously demonstrated that even in the absence of a host systemic inflammatory/immune response to the parasite, ventricular arrhythmias can be induced by Lister 427 trypanosomes *ex vivo* due to the effect of a secreted/excreted protease, *T*. *brucei* cathepsin-L (TbCatL), on cardiomyocytes [24]. However, no significant ECG abnormalities were apparent between the control and infected animals *in vivo*. Four explanations may account for this. First is the use of anaesthesia in the Lister 427 model compared with conscious ECG monitoring in the TREU 927 model. It is possible that the anaesthesia (isofluorane) used in the Lister 427 model reduced the propensity for arrhythmias, but to the best of our knowledge there is little evidence that isofluorane can reduce the incidence of arrhythmias. Secondly, the brevity of the infection period may limit appreciable extravasation of the parasite into the cardiac extracellular matrix. This possibility is supported by histological examination of Lister 427 infected hearts, in which no parasites could be identified in the interstitium, suggesting that extravasation had not occurred with this strain—at least within the limits of detection. Connected to the brevity of the infection period, exposure time of cardiomyocytes to parasites and parasite products in the Lister 427 model is clearly much less than that of the TREU 927 model. However, it is important to note that the parasite burden within Lister 427 infected animals is much greater than the TREU 927 model (2.51 x 10^8^ ± 1.02 x 10^8^ parasites.mL^-1^ compared to 3.56 x 10^7^ ± 1.62 x 10^7^ parasites.mL^-1^, respectively, at peak parasitaemia). On balance therefore it is difficult to conclude that the brevity of infection period leading to reduced exposure time of cardiomyocytes to parasites and parasite products exclusively explains an absence of arrhythmias in the 427 infection model. Thirdly, Lister 427 does not readily extravasate without existing tissue damage from an inflammatory processes [36]. In the current study, histological examination confirmed that the level of inflammation in Lister 427 hearts was not significantly different from that in control hearts. Finally, it is probable that the amount of TbCatL produced by Lister 427 is significantly lower than that produced by pleomorphic trypanosomes such as TREU 927. Caffrey *et al*. (2001) demonstrated that short-stumpy form trypanosomes produce almost five times the amount of TbCatL compared to long-slender forms [37]. Since the Lister 427 strain does not differentiate and remains as a long-slender form, the quantity of TbCatL produced is very likely to be lower and contribute to the lack of response observed.

### HR Changes in *Ex Vivo* Hearts from *T*. *b*. *brucei* Lister 427 Infected Animals

During pathophysiological stress, there are increased levels of circulating catecholamines (adrenaline and noradrenaline). In a previous study, *ex vivo* hearts demonstrated a significantly higher frequency of ventricular arrhythmias (ventricular premature complexes, VPCs) in response to trypanosome culture supernatant and isoproterenol (ISO; a β-adrenergic agonist) [24]. This effect was mediated by a CaMKII-dependent increase of spontaneous sarcoplasmic reticulum (SR)-mediated Ca^2+^ release events (Ca^2+^ waves) [24;38]. In the current study, *ex vivo* hearts from the Lister 427 infection model demonstrated no increase in arrhythmic events but a significantly increased HR in response to ISO. HR is dictated by the rate that sinoatrial nodal pacemaker cells (SANC) in the heart fire spontaneous action potentials. It is possible that the Lister 427 infection model induces a higher HR due to: 1) a greater sensitivity of the sinoatrial nodal pacemaker cells (SANC) to β-adrenergic stimulation as can happen in heart failure [39]; 2) altered expression/heterodimerisation/polymorphisms of β_1_ adrenergic receptors [40;41] in SANC; 3) altered expression of ion channels governing SANC pace making such as hyperpolarisation and cyclic nucleotide (HCN) channels [42], G-protein-activated inwardly rectifying potassium (GIRK) channels [43] or calcium channels [44]; 4) altered rate of SANC action potential firing due to changes in Ca^2+^ handling protein activity [44;45]. CaMKII-mediated phosphorylation of Ca^2+^ handling proteins may alter HR via modulation of the rate of action potential firing from SANCs [45]. The possibility that the observed HR change in the current study may be a CaMKII-mediated effect on Ca^2+^ handling in SANCs in response to trypanosomes warrants future investigation. The implications of a stress-induced increased HR during trypanosome infection are important as this may in turn lead to an increased metabolic demand on the heart at the expense of the other organs [46;47]. Given that patients with AT can die from multiple organ failure [32] this increased metabolic demand is potentially significant.

### *Ex Vivo* ECG Parameters for *T*. *b*. *brucei* Lister 427 Infections

The PR interval was not significantly altered by infection with Lister 427, but tended to decrease at 1 μM ISO in control animals while remaining unchanged in infected animals. A failure for the PR interval to shorten under β-adrenergic stimulation may reflect a decreased ability of atrio-ventricular (AV) node conduction velocity to respond to β-adrenergic stimulation (1^st^ degree AV block) [48]. A PR interval of >200 ms has been reported in 3.7–14% of HAT patients and was defined as 1^st^ degree AV block [15;18]. However, a separate study Blum *et al*. (2007) did not observe a significant change in PR interval in patients with HAT [19]. It is therefore unclear whether trypanosome infection consistently results in altered AV node conduction velocity.

The QTc was not significantly affected by infection with Lister 427. A prolongation of QTc during clinical infection may be attributed to myocardial inflammatory infiltration leading to altered electrical conductivity [9;12]. The cause of the QTc prolongation identified by Blum *et al*. (2007) was not investigated but ascribed to the historical observation of myocardial inflammatory infiltration in patients with HAT [19].

### *In Vivo* ECG Parameters for *T*. *b*. *brucei* TREU 927 Infections

The pleomorphic strain *T*. *b*. *brucei* TREU 927 was used to more closely approximate a natural infection. This TREU 927 infection model demonstrated the classical undulating parasitaemia phenotype (Fig 3B) and was sustainable over a longer time period (up to 11 days) compared to the four-day infection model of Lister 427. Implanted biopotential recording devices had the additional advantage of avoiding any cardio-depressive effect of anaesthesia while assessing ECG parameters. In parallel with the Lister 427 infection model, the PR and QTc interval was not significantly altered in the TREU 927 infection model. However, the normalised HR of the TREU 927 infected animals *in vivo* was significantly increased. It is unknown whether the anaesthetic regime used during ECG assessment in the Lister 427 infection model masked an increased HR. Interestingly, a significant increase in the frequency of ventricular arrhythmias (VPCs) with the TREU 927 infection model was also observed *in vivo*. The change in arrhythmia frequency increased over the course of the model independent of the parasitaemia level since the arrhythmia frequency remained increased when the parasitaemia reduced after the first peak. This provides further evidence that African trypanosomes increase the propensity for ventricular arrhythmias *in vivo*.

### HR Changes in *Ex Vivo* Hearts from *T*. *b*. *brucei* TREU 927 Infections

As with the Lister 427 infection model, increasing concentrations of ISO elevated HR to a greater degree in TREU 927 infected hearts. This supports an effect of trypanosome infection on the response of the SANCs to ISO. Interestingly, HR *in vivo* was significantly increased in the TREU 927 infection model (Fig 3C(i)), an effect lost upon removal of the heart *ex vivo*, but reintroduced with the addition of ISO. It is possible that a circulating factor (which ISO simulates) underlies the effect on HR observed in the TREU 927 infection model *in vivo*.

### *Ex Vivo* ECG Parameters for *T*. *b*. *brucei* TREU 927 Infections

In parallel with the Lister 427 infection model, the PR interval tended to decrease in control animals with a significant decrease in control animals at 1 μM ISO compared to no ISO, and was significantly reduced compared to PR intervals in infected animals at 0.1, 1 and 10 μM ISO. Although this suggests an effect on AV node conduction velocity similar to that reported in a proportion of HAT cases [15;18], the lack of PR interval effect in rats *in vivo* suggests that alteration of AV node conduction velocity may not be a significant feature in the rat *in vivo* model of trypanosome infection. The QTc was not significantly altered despite an increase in the level of inflammation (Fig 6B(ii)). It may be possible that the levels of inflammation in the TREU 927 infection model were insufficient to induce a prolongation of the QTc interval as observed in patients with HAT, which is understandable given that HAT is a chronic disease of months to years. However, it should be noted that direct comparison of QT intervals between rats and humans should be interpreted with care since different channels are responsible for QT interval control in these species [49].

### *T*. *b*. *brucei* TREU 927 Infected *Ex Vivo* Hearts Show an Increased Frequency of Arrhythmias

Increased VPC frequency was observed in the TREU 927 infection model *in vivo* in parallel with increased levels of inflammation and the presence of extravasated parasites. However, VPCs were absent when the same hearts were isolated *ex vivo*, only re-emerging in the presence of ISO. These novel data suggest that African trypanosomes create an arrhythmogenic substrate in the heart, which when triggered by circulating factor(s) increases the propensity for ventricular arrhythmias. The generation of an arrhythmogenic substrate in the current study is not caused by a significant increase of fibrosis (a known arrhythmogenic factor) in the TREU 927 infection model (Fig 6). However, the increased levels of inflammation observed and the ability of *T*. *b*. *brucei* TREU 927 to extravasate into the myocardium—both of which occur despite the parasitaemia levels being approximately 7 fold lower than with *T*. *b*. *brucei* Lister 427—are likely contributors to the arrhythmogenic substrate. Importantly, the *ex vivo* data reveal that these two factors by themselves are not able to lead to arrhythmias without an additional circulating trigger. It is possible that β-adrenergic stimulation such as circulating adrenaline/noradrenaline *in vivo* is the trigger for ventricular arrhythmias during trypanosome infection and is mimicked by ISO when applied to *ex vivo* hearts from infected animals. Whether the expected elevated levels of trypanosome-secreted TbCatL (compared with the Lister 427 infection model) *in vivo* contribute to the trigger *via* increased CaMKII activity (as would occur with β-adrenergic stimulation [50]) remains unknown and warrants further investigation in future studies.

### Study Limitations

There are key differences in the infection profiles of the monomorphic and pleomorphic infection models that make direct comparison between the models difficult. The exponential nature of parasite multiplication in the monomorphic Lister 427 model removes the ability to examine infections beyond the first peak of parasitaemia. This biological limitation also results in a lack of representation of parasite life cycle stages (i.e. short stumpy trypanosomes) relevant to infections in the field, resulting in restricted utility of the monomorphic model for exploring cardiac pathogenesis in a meaningful manner. The novel data therefore highlight the requirement for infection models that are capable of reflecting the chronic infection profiles of HAT and AAT infections (e.g. pleomorphic TREU 927) to study HAT-induced cardiac pathophysiology.

### Conclusion

The current study has successfully characterised *in vivo* cardiac electrical dysfunction in two infection models of African trypanosomes. We have demonstrated for the first time that infection with a pleomorphic strain of African trypanosome produces an increased frequency of ventricular arrhythmias and provide evidence that this phenomenon requires a circulating factor in combination with trypanosome infection. This novel animal model of African trypanosomiasis provides a highly attractive platform to further study the cardiac/other organ-related pathophysiology of African trypanosomiasis and the efficacy of therapeutic strategies to treat this neglected disease.

## Supporting Information

S1 Fig Organ mass data for *T*. *b*. *brucei* Lister 427 infection model. (A(i)) Mean ± SEM for body mass (302.7 ± 4.1 *vs.* 301.0 ± 3.1 g; control (*n* = 24) *vs*. infected (*n* = 21); *P*>0.05). (ii-iv) Mean ± SEM for organ mass to tibial length ratio for heart (29.3 ± 2.7 *vs.* 27.8 ± 4.8 mg.mm^-1^; control *vs*. infected; *P*>0.05), liver (226.5 ± 5.7 *vs.* 246.2 ± 8.7 mg.mm^-1^; control *vs*. infected; *P*>0.05), lung (33.1 ± 2.6 *vs.* 31.4 ± 1.9 mg.mm^-1^; control *vs*. infected; *P*>0.05) and spleen (13.3 ± 0.4 *vs.* 26.5 ± 3.4 mg.mm^-1^; control *vs*. infected; *P*<0.05). (B) Photograph of splenic enlargement.
(TIF)

S2 Fig Organ mass data for *T*. *b*. *brucei* TREU 927 infection model. (A(i)) Mean ± SEM for body mass (344.0 ± 8.5 *vs.* 314.7 ± 15.3 g; control (*n* = 5) *vs*. infected (*n* = 5); *P*>0.05). (ii-iv) Mean ± SEM for organ mass to tibial length ratio for heart (34.1 ± 3.4 *vs.* 31.8 ± 5.6 mg.mm^-1^; control vs. infected; *P*>0.05), liver (253.3 ± 8.9 *vs.* 244.7 ± 17.6 mg.mm^-1^; control *vs*. infected; *P*>0.05) and spleen (17.2 ± 1.6 *vs.* 50.2 ± 7.5 mg.mm^-1^; control *vs*. infected; *P*<0.05).
(TIF)
