# Supplementary figures and images for: A Pre-clinical Animal Model of Trypanosoma brucei Infection Demonstrating Cardiac Dysfunction
Source: PLoS Negl Trop Dis. 2015 May 29;9(5):e0003811. doi: 10.1371/journal.pntd.0003811 (PMC4449042; doi:10.1371/journal.pntd.0003811)

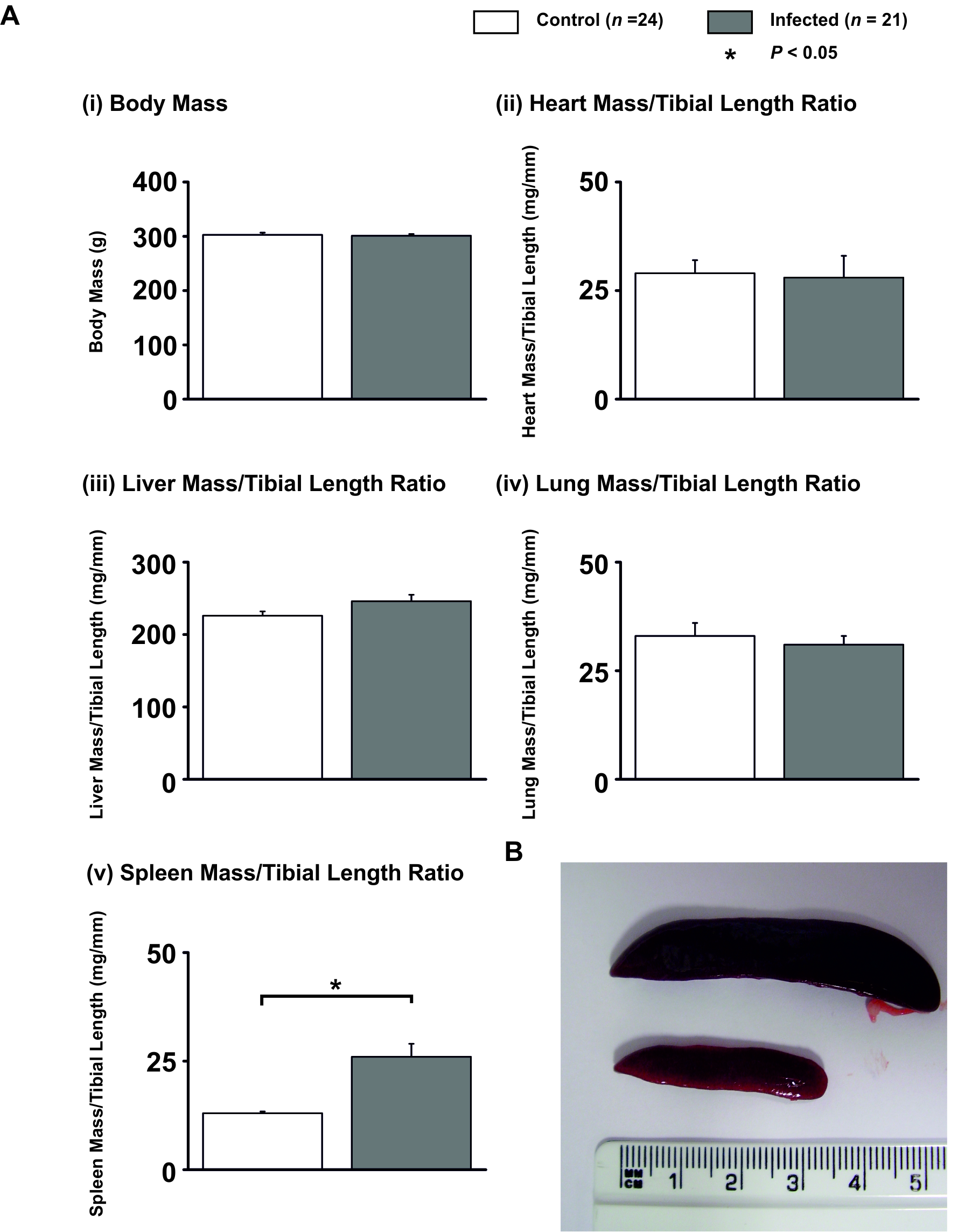

Supplement: S1 Fig — (A(i)) Mean ± SEM for body mass (302.7 ± 4.1 vs. 301.0 ± 3.1 g; control (n = 24) vs. infected (n = 21); P>0.05). (ii-iv) Mean ± SEM for organ mass to tibial length ratio for heart (29.3 ± 2.7 vs. 27.8 ± 4.8 mg.mm-1; control vs. infected; P>0.05), liver (226.5 ± 5.7 vs. 246.2 ± 8.7 mg.mm-1; control vs. infected; P>0.05), lung (33.1 ± 2.6 vs. 31.4 ± 1.9 mg.mm-1; control vs. infected; P>0.05) and spleen (13.3 ± 0.4 vs. 26.5 ± 3.4 mg.mm-1; control vs. infected; P<0.05). (B) Photograph of splenic enlargement. (TIF) [file pntd.0003811.s001.tif]

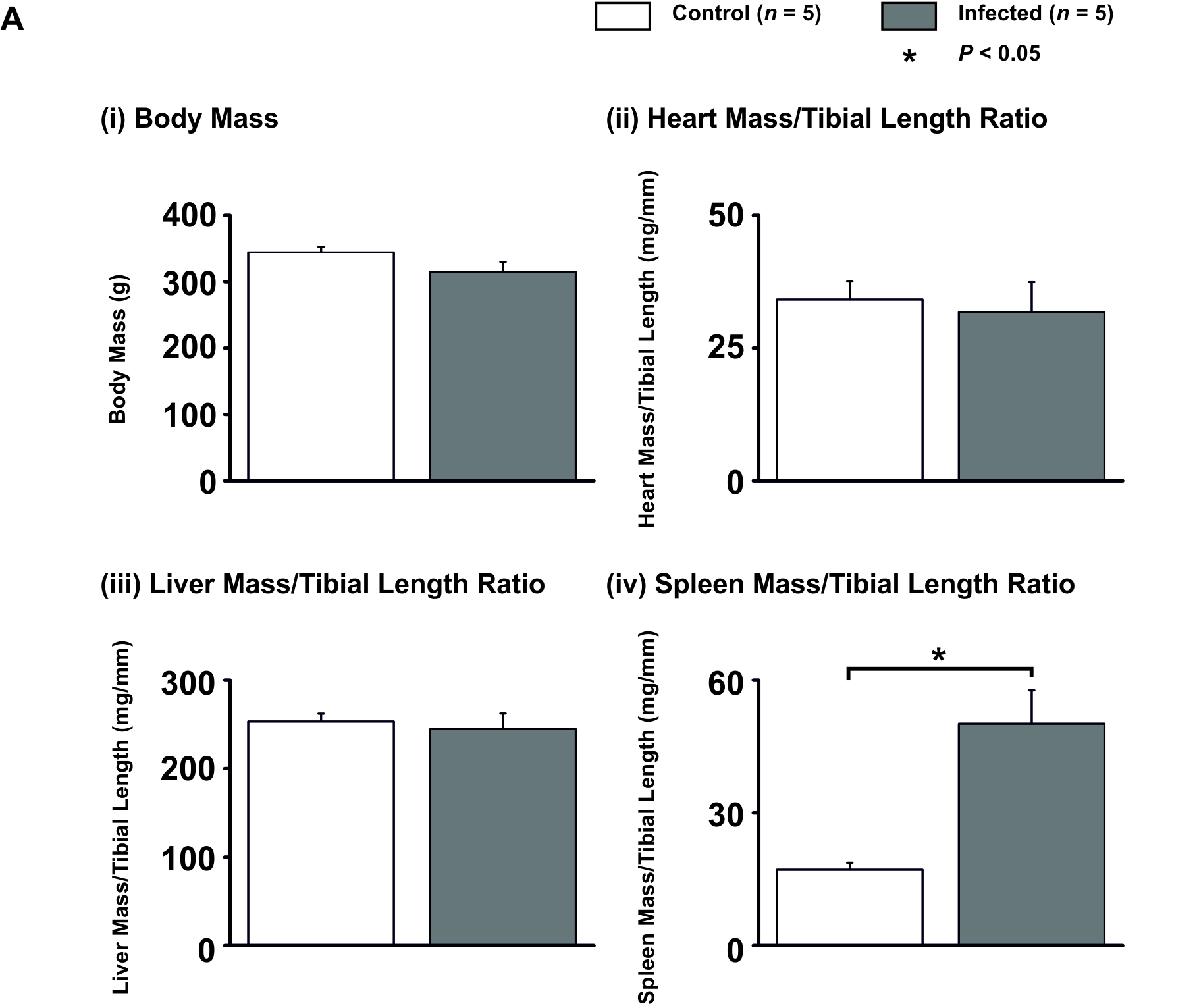

Supplement: S2 Fig — (A(i)) Mean ± SEM for body mass (344.0 ± 8.5 vs. 314.7 ± 15.3 g; control (n = 5) vs. infected (n = 5); P>0.05). (ii-iv) Mean ± SEM for organ mass to tibial length ratio for heart (34.1 ± 3.4 vs. 31.8 ± 5.6 mg.mm-1; control vs. infected; P>0.05), liver (253.3 ± 8.9 vs. 244.7 ± 17.6 mg.mm-1; control vs. infected; P>0.05) and spleen (17.2 ± 1.6 vs. 50.2 ± 7.5 mg.mm-1; control vs. infected; P<0.05). (TIF) [file pntd.0003811.s002.tif]
